# Protein refolding in peroxisomes is dependent upon an HSF1-regulated function

**DOI:** 10.1007/s12192-012-0335-5

**Published:** 2012-04-05

**Authors:** Lonneke Heldens, Siebe T. van Genesen, Lars L. P. Hanssen, Jurre Hageman, Harm H. Kampinga, Nicolette H. Lubsen

**Affiliations:** 1 271 Department of Biomolecular Chemistry, Radboud University Nijmegen, P.O. Box 9101, 6500 HB Nijmegen, The Netherlands; 2 Section of Radiation and Stress Cell Biology, Department of Cell Biology, University Medical Center Groningen, University of Groningen, Groningen, 9700 AD The Netherlands

**Keywords:** Chaperone network, Heat shock proteins, Cellular stress response, Intra-organellar refolding, Protein denaturation, Thermotolerance

## Abstract

Post-heat shock refolding of luciferase requires chaperones. Expression of a dominant negative HSF1 mutant (dnHSF1), which among other effects depletes cells of HSF1-regulated chaperones, blocked post-heat shock refolding of luciferase targeted to the cytoplasm, nucleus, or peroxisomes, while refolding of endoplasmic reticulum (ER)-targeted luciferase was inhibited by about 50 %. Luciferase refolding in the cytoplasm could be partially restored by expression of HSPA1A and fully by both HSPA1A and DNAJB1. For full refolding of ER luciferase, HSPA1A expression sufficed. Neither nuclear nor peroxisomal refolding was rescued by HSPA1A. A stimulatory effect of DNAJB1 on post-heat shock peroxisomal luciferase refolding was seen in control cells, while refolding in the cytoplasm or nucleus in control cells was inhibited by DNAJB1 expression in the absence of added HSPA1A. HSPB1 also improved refolding of peroxisomal luciferase in control cells, but not in dnHSF1 expressing cells. HSP90, HSPA5, HSPA6, and phosphomevalonate kinase (of which the synthesis is also downregulated by dnHSF1) had no effect on peroxisomal refolding in either control or chaperone-depleted cells. The chaperone requirement for post-heat shock refolding of peroxisomal luciferase in control cells is thus unusual in that it can be augmented by DNAJB1 or HSPB1 but not by HSPA1A; in dnHSF1 expressing cells, expression of none of the (co)-chaperones tested was effective, and an as yet to be identified, HSF1-regulated function is required.

**Electronic supplementary material:**

The online version of this article (doi:10.1007/s12192-012-0335-5) contains supplementary material, which is available to authorized users.

## Introduction

Proteins are made as linear chains but are active as intricately folded units often associated in larger assemblies. In dilute solution in vitro, many proteins can fold without help, but in the in vivo high protein concentration environment proteins need help from molecular chaperones which prevent protein aggregation and provide a folding surface. Molecular chaperones also bind unfolded proteins and either help these to refold or target these for degradation by the proteasome or via autophagy (Balch et al. 2008). Cells contain an unknown but probably large number of chaperones dedicated to the folding of a single protein or assembly of a single complex (see, for example, Christis et al. 2008). The three main cellular compartments, cytoplasm, mitochondria, and ER, each have a compartment dedicated general chaperoning network. These networks are similar in that they all contain related chaperones and associated factors which aid in folding (Hsp90 and Hsp70 machines; Welch and Feramisco 1982; Mayer and Bukau 2005), chaperones which deliver substrates to the folding machines (DNAJ/Hsp40 proteins), and chaperones which can store unfolded protein for later refolding or degradation (the small heat shock proteins) (Vos et al. 2008). For example, HSPA5 (GRP78, BiP) is the ER-specific Hsp70 paralog and GRP94 the ER Hsp90 paralog (Lin et al. 1993), while HSPA9 (Bhattacharyya et al. 1995) and TRAP1 (Landriscina et al. 2010) are the mitochondrial Hsp70 and Hsp90 paralogs. The nuclear compartment does not have its own chaperoning network. Nuclear proteostasis mostly relies on transient passage of cytoplasmic chaperones (Velazquez and Lindquist 1984; Ohtsuka and Laszlo 1992), and the nucleus is a poor folding environment (Michels et al. 1995; Hageman et al. 2007). Peroxisome-specific chaperones have been found in plant (Corpas and Trelease 1997; Ma et al. 2006) but not in animal cells. In some, but not all (Kikuchi et al. 2004; Schlüter et al. 2007), studies chaperones were detected in the peroxisome proteome (Mi et al. 2007; Wiese et al. 2007), but it cannot be excluded that these were trace contaminants as these were either cytoplasmic, ER, or mitochondrial proteins. Peroxisomes are thought to bud off from the ER, but their matrix enzymes are imported from the cytoplasm (Gould and Collins 2002). Protein import in the peroxisome is unusual in that fully folded proteins and even oligomeric protein complexes can be imported (McNew and Goodman 1996). Peroxisomal matrix proteins could thus be folded in the cytoplasm, and no chaperoning would then be required in the peroxisomal matrix. However, the high peroxisomal production of ROS (Zwacka et al. 1994), together with the discovery of several ROS-metabolizing enzymes in peroxisomes (De Duve and Baudhuin 1966; Elliott et al. 1986; Zwacka et al. 1994; Stolz et al. 2002), suggests that peroxisomes have to cope with oxidative stress, which makes it peculiar that no chaperones are found within this organelle. In spite of the lack of classical chaperones, heat-denatured peroxisomal luciferase is refolded as efficiently as it is in other cellular compartments (Hageman et al. 2007), suggesting that peroxisomes can deal with unfolded proteins.

The chaperoning capacity of the various cellular compartments can be augmented by additional synthesis of chaperones when proteostasis in that compartment is sensed to fail. Mitochondria have an unfolded protein response, also referred to as UPR^mt^ (Haynes and Ron 2010), as does the ER (UPR^ER^; Ron and Walter 2007). Unfolding proteins in the cytoplasm result in the activation of heat shock factor 1 (HSF1) and the increased transcription of the HSF1 target genes, among which those encoding cytoplasmic chaperones, the heat shock proteins. The result is a temporary increase in the chaperoning capacity allowing the cells to deal with unfolded proteins either by refolding or degradation. The higher level of chaperones also provides protection to a second proteotoxic insult. Heat shocking a cell thus provides it with thermotolerance, i.e., resistance to a second heat shock (McMillan et al. 1998; Xiao et al. 1999; Pirkkala et al. 2000; Zhang et al. 2002). Such tolerance can be induced in all compartments, including the peroxisomes (Hageman et al. 2007), even though the existence of a UPR^per^ has not been demonstrated.

We have previously shown that even under physiological conditions and at normal growth temperatures (i.e., without stress), HSF1 regulates the level of cytoplasmic chaperones. Expression of a dominant negative mutant of HSF1 in HEK293 cells depletes cells of the classical HSF1-regulated chaperones such as HSP90AA1 (Hsp90), HSPA1A (Hsp70), DNAJB1 (Hsp40), and HSPB1 (Hsp27) (Heldens et al. 2010). By exogenous expression of single chaperones, it can then be tested which chaperone is limiting for the folding/activity of a particular chaperone client. Besides providing a fundamental insight in the substrate specificity and the critical nodes of the protein folding network of the cell, such information is of interest as HSF1 loses its activity during aging, making the aging cell prone to protein folding disease (Fawcett et al. 1994; Heydari et al. 2000). Increasing expression of a (co-)chaperone would be a way of restoring proteostasis.

A common way to probe the chaperoning capacity of a cellular compartment is to measure the refolding of heat denatured luciferase targeted to that compartment (Hageman et al. 2007). In control cells, luciferase can be equally well refolded in the cytoplasm, the ER, and the peroxisome, while the nucleus is a poor refolding environment. In vivo refolding of luciferase, at least in the cytoplasm, requires the Hsp70 folding machine (Nollen et al. 1999) and can be increased by exogenous expression of HSPA1A (Hageman et al. 2007; Michels et al. 1997), HSPA1A and DNAJB1 (Michels et al. 1997), and HSPB1 (Bryantsev et al. 2007). We show here that expression of dnHSF1 affects the luciferase refolding capacity not only in the cytoplasm or the nucleus but also in the peroxisomes and the ER. We further show that only in the peroxisomes refolding cannot be restored by exogenous expression of HSPA1A and DNAJB1. Intriguingly, peroxisomal refolding seems to be sensitive to changes in expression of HSPB1, a cytoplasmic protein.

## Materials and methods

### Western blot analysis

Cell pellets were homogenized in buffer containing 50 mM Tris–HCl pH 7.5, 150 mM NaCl, 1 % Triton X-100, 100 mM NaF, 20 mM Na_4_P_2_O_7_, 1 mM phenylmethylsulfonyl fluoride, and protease inhibitors (Complete Mini; Roche). Then 4× sample buffer (200 mM Tris–HCl 6.8, 20 % β-mercaptoethanol, 8 % sodium dodecyl sulfate, 40 % glycerol, and 0.4 % bromophenol blue) was added and the lysates were incubated at 95°C for 5 min. Protein samples were separated in 10 % polyacrylamide gels and transferred to nitrocellulose transfer membrane (Protran) using a Bio-Rad Mini-PROTEAN II Electrophoresis cell according to the manufacturer’s instructions. For western blot analysis, polyclonal HSF1 antibody (SPA-901; Stressgen) was used at a 1: 1,000 dilution; monoclonal HSP90 antibody (610418, BD Biosciences) at a 1:1,000 dilution; polyclonal HSPB1 antibody, obtained from Dr. A. Zantema, at a dilution of 1:400; monoclonal HSPA1A antibody 4 G4 (ab5444; Abcam) at a 1:5,000 dilution; polyclonal HSPA5 antibody, kindly donated by Prof. Dr. Ineke Braakman, at a dilution of 1:1,000; polyclonal DNAJB1 antibody (SPA-400; Stressgen) at a 1:10,000 dilution; polyclonal phosphomevalonate kinase (PMVK) antibody, obtained from Dr. H.R. Waterham (Hogenboom et al. 2002), at a 1:500 dilution; monoclonal V5 antibody (R96025; Invitrogen) at a 1:5,000 dilution; polyclonal γ-tubulin antibody (GTU-88; Abcam) at a 1:1,000 dilution; and monoclonal β-actin antibody (AC-15, Sigma-Aldrich) at a dilution of 1:5,000. Blots were incubated with fluorescent secondary antibodies IRDye® 800CW conjugated goat (polyclonal) anti-rabbit IgG and IRDye® 800CW conjugated goat (polyclonal) anti-mouse IgG (926-32211 and 926-32210, respectively; LI-COR Biosciences) according to the manufacturer’s instructions and scanned using a LI-COR Odyssey infrared scanner. Signals were quantified using Odyssey version 2.1 software.

### Cell culture

Flp-In T-REx-293 cells were manipulated according to the manufacturer’s instructions using the T-REx system (Invitrogen). The stable cell lines HEK-dnHSF1 and HEK-cDNA5 have been described previously (Heldens et al. 2010). Flp-In T-REx-293 cells stably transfected with Per-superluc-eGFP selected with 0.5 mg/ml Geneticin (Invitrogen) were described previously as well (Hageman et al. 2007). These cells were in addition stably transfected with the tetracycline-inducible plasmids pcDNA5-dnHSF1 or pcDNA5-FRT/TO to yield HEK-dnHSF1-Per-superluc-eGFP and HEK-cDNA5-Per-superluc-eGFP. Cells were cultured at 37°C/5 % CO_2_ in high-glucose DMEM medium supplemented with 10 % fetal calf serum and 100 U/ml penicillin and 100 μg/ml streptomycin. Blasticidin (1.65 μg/ml; Invitrogen) and 100 μg/ml hygromycin were also added to the culture medium during maintenance of the cell lines but were omitted during experiments. Cell lines stably transfected with Per-superluc-eGFP were also cultured with Geneticin (0.5 mg/ml).

### Recombinant DNA constructs

The C-terminal truncation mutant of dnHSF1 containing codons 1-379 from HSF1 as well as pcDNA5-HSPB1 and pcDNA5-HSP90AA1 were described earlier (Heldens et al. 2010). The plasmids pcDNA5-V5-DNAJB1 and pcDNA5-V5-HSPA1A, pcDNA5-V5-HSPA6, Cyt-superluc-eGFP, ER-superluc-eGFP, and Nuc-superluc-eGFP were described in Hageman et al. (2007, 2011). pcDNA5-PMVK was made by PCR amplifying the cDNA from HEK293 RNA using the primers PMVK up 5′-agctaagcttagtggccgcgtccat-3′ and PMVK low 5′-cctcagaatctagacccccc-3′ and cloning the PCR fragment (HindIII-XbaI(bl)) into pcDNA5-FRT/TO (HindIII-XhoI(bl)).

### Transfections and reporter gene assays

Transient transfections were performed using FuGENE-6 (Roche) according to the manufacturer’s instructions. Cells were seeded on 24-well plates and on the next day transfected with 0.2 μg plasmid per well.

For analysis of the refolding capacity in different organelles, cells were transfected with a mixture of 10 ng luciferase reporter plasmid and 30 ng β-actin-β-galactosidase and 160 ng expression plasmids. To transfect equal amounts of expression plasmid, transfections with one chaperone coding expression plasmid were supplemented with an empty vector. In experiments using HEK-Per-superluc-eGFP, 40 ng β-actin–β-galactosidase was used because no additional luciferase reporter was needed. Heat shock was performed at 45°C for 30 min. Cells were harvested after a recovery period of 1, 2, or 3 h at 37°C as indicated. Cycloheximide (0.2 mg/ml) was added prior to heat shock to block de novo synthesis of luciferase. Cells were lysed in 200 μl reporter lysis mix (25 mM Bicine, 0.05 % Tween 20, 0.05 % Tween 80) for 10 min. For the β-galactosidase assay, 10 μl cell lysate was mixed with 100 μl Galacton solution (100 mM Na-phosphate pH 8.2, 10 mM MgCl_2_, 1 % Galacton-Plus; Tropix). After 30 min incubation at room temperature, 150 μl accelerator II (Tropix) was added and luminescence was measured with the Lumat LB 9507 tube luminometer (Berthold). For the luciferase assay, 10 μl cell lysate was mixed with 50 μl luciferin solution, and luminescence was again measured with the Lumat luminometer. Data shown are the mean ± SD of two or more independent experiments with four replicates each. The data points used to describe the refolding capacity of an organelle were obtained through subtraction of the luciferase activity directly after heat shock. These values varied between 0.1 % for nuclear luciferase and 15 % for ER luciferase and are presented in S1–S3.

## Results

### Experimental design

To measure the chaperoning capacity in the nucleus, cytoplasm, or ER, we used expression constructs for luciferase–eGFP targeted to these different compartments (Hageman et al. 2007) transiently transfected into HEK293 cells with or without expression of dominant negative HSF1. Transient transfection of an expression construct for luciferase–eGFP with a peroxisomal targeting sequence leads to mislocalization (Jankowski et al. 2001; Hageman et al. 2007). To monitor refolding in peroxisomes, we therefore used a cell line stably transfected with both the peroxisomal-targeted luciferase–eGFP expression construct (Hageman et al. 2007) and the tetracycline inducible dnHSF1 expression construct. In pilot experiments, we found that expression of dnHSF1 needed to be induced for at least 48 h to see a decrease in the post-heat shock refolding of cytoplasmic luciferase. Thus, when cells expressing dnHSF1 were used, refolding was measured 96 h after induction of dnHSF1 expression. The fact that there is a delayed response to induction of dnHSF1 indicates that it is not the expression of dnHSF1 itself that leads to a reduced refolding capacity but that it is a secondary effect of the expression of dnHSF1, such as chaperone depletion. The amount of active luciferase remaining in cells harvested directly after heat shock varied between 1 and 10 % of the pre-heat shock value depending on the cellular compartment and the chaperone complement (Fig. S1). In the data presented below, only the rate of refolding under the various experimental conditions is presented.

### Effect of dnHSF1 expression on luciferase refolding in different cellular organelles

**Fig. 1:**
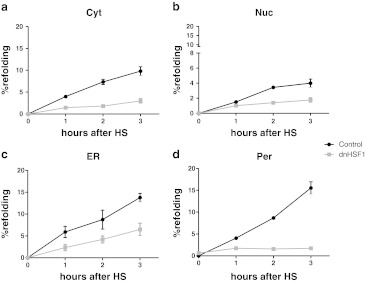
Effect dnHSF1 on refolding capacities of different organelles. Relative luciferase activity of **a** Cyt-superluc-eGFP in HEK-cDNA5 and HEK-dnHSF1 cells; **b** Nuc-superluc-eGFP in HEK-cDNA5 and HEK-dnHSF1 cells; **c** ER-superluc-eGFP in HEK-cDNA5 and HEK-dnHSF1 cells; **d** Per-superluc-eGFP in HEK-cDNA5-Per-superluc-eGFP or HEK-dnHSF1-Per-superluc-eGFP cells. Cells were harvested after heat shock at the times indicated, and the luciferase activity was measured. The luciferase activity shown is relative to the activity in non-heat-shocked cells transfected and cultured in parallel. The results are the average of four independent transfections (standard deviations are indicated by *error bars*)

**Fig. 2:**
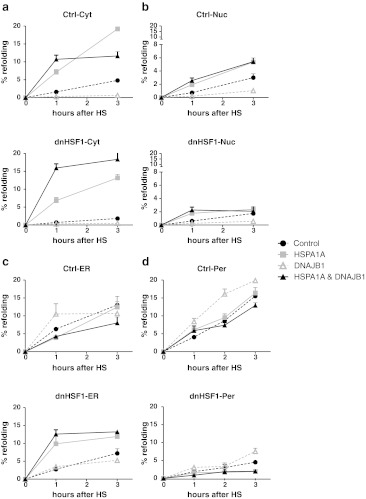
Effect dnHSF1 and HSPA1A or DNAJB1 on refolding capacities of different organelles. Relative luciferase activity of **a** Cyt-superluc-eGFP in HEK-cDNA5 and HEK-dnHSF1 cells; **b** Nuc-superluc-eGFP in HEK-cDNA5 and HEK-dnHSF1 cells; **c** ER-superluc-eGFP in HEK-cDNA5 and HEK-dnHSF1 cells; **d** Per-superluc-eGFP in HEK-cDNA5-Per-superluc-eGFP or HEK-dnHSF1-Per-superluc-eGFP cells. Cells were co-transfected with the luciferase reporter gene and expression constructs for HSPA1A and/or DNAJB1 and/or empty vector as indicated. Per-superluc-eGFP cells were transfected only with the expression constructs. Expression levels of exogenously expressed proteins are shown in Fig. S5. Cells were harvested after heat shock at the times indicated, and the luciferase activity was measured. Relative luciferase activities were calculated as detailed in the legend to Fig. 1. The results are the average of four independent transfections (standard deviations are indicated by *error bars*)

Under our experimental conditions, between 10 % (cytoplasm) and 15 % (ER, peroxisomes) of the luciferase was refolded 3 h post-heat shock in control cells. In the nucleus, refolding was far less efficient and after 3 h only 4 % of the luciferase was refolded (Fig. 1). As expected, dnHSF1 expression severely inhibited post-heat shock luciferase refolding in the cytoplasm and nucleus. Unexpectedly, peroxisomal luciferase was also not refolded in dnHSF1 expressing cells. Refolding of ER-targeted luciferase was less sensitive to dnHSF1 expression and was inhibited by about 50 % (Fig. 1). Luciferase refolding is known to be mediated by the hsp70 folding machinery, and refolding of cytoplasmic luciferase in dnHSF1 expressing cells could be completely restored by exogenous expression of both HSPA1A and DNAJB1. In control cells, there was no synergistic effect between HSPA1A and DNAJB1 and expression of just DNAJB1 even inhibited refolding (Fig. 2a), as previously reported (Michels et al. 1997). The refolding of nuclear luciferase in dnHSF1 expressing cells was not significantly improved by expression of either HSPA1A, DNAJB1, or both, while HSPA1A or HSPA1A and DNAJB1 did increase refolding of nuclear luciferase in control cells (Fig. 2b). DNAJB1 again inhibited. Exogenous expression of HSPA1A, DNAJB1, or both had little effect on the refolding of ER-targeted luciferase in control cells, but HSPA1A did restore ER luciferase refolding in dnHSF1 expressing cells. Exogenous expression of DNAJB1 had no effect. ER resident DNAJ proteins, which are not HSF1 regulated, might take the role of DNAJB1 in the ER. Unlike ER luciferase, refolding of peroxisomal luciferase in dnHSF1 expressing cells could not be restored by exogenous expression of HSPA1A. The peroxisomal compartment was the only compartment in which exogenous expression of DNAJB1 had a stimulatory effect on refolding, but this effect was only significant in control cells and not in cells expressing dnHSF1 (Fig. 2d). These data show that HSF1-regulated gene products do contribute significantly to the chaperoning capacity (as measured by post-heat shock luciferase refolding) of all cellular compartments, including the ER and the peroxisomes. However, the critical nodes in the various compartments differ: HSPA1A restored the folding deficiency in dnHSF1 expressing cells in the cytosol and ER but did not so in nuclei or peroxisomes. The properties of the peroxisomal refolding network are unique in that it is the only compartment in which exogenous expression of DNAJB1 stimulated rather than inhibited refolding (in control cells).

The effect of chaperone depletion on refolding in ER and peroxisomes could be indirect: A deficit in cytoplasmic chaperoning capacity could have as a secondary effect the overloading of the ER and peroxisomal refolding machinery. We therefore tested if exogenous expression of HSPA5, an abundant ER chaperone, could compensate for depletion of the HSF1-regulated chaperones. Exogenous expression of HSPA5 had a significant effect on the thermostability of luciferase in all compartments, except the nucleus, as it about doubled the yield of luciferase activity directly after heat shock (data not shown). Curiously, this protective effect during heat shock did not correlate with an improvement in refolding after heat shock: Exogenous expression of HSPA5 actually inhibited refolding in all compartments (data not shown).

### Thermotolerance of peroxisomal refolding is dependent upon HSF1-regulated gene products

**Fig. 3:**
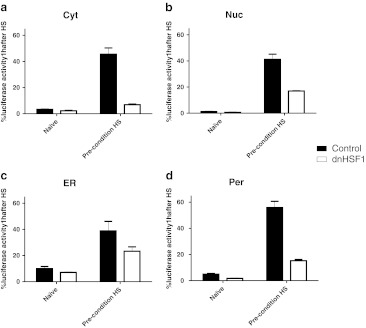
HSF1 dependent thermotolerance in different organelles. Thermotolerance in different cellular compartments. **a** Cyt-superluc-eGFP in HEK-cDNA5 and HEK-dnHSF1 cells; **b** Nuc-superluc-eGFP in HEK-cDNA5 and HEK-dnHSF1 cells; **c** ER-superluc-eGFP in HEK-cDNA5 and HEK-dnHSF1 cells; **d** Per-superluc-eGFP in HEK-cDNA5-Per-superluc-eGFP or HEK-dnHSF1-Per-superluc-eGFP cells. Cells were either given a pre-heat shock at 45°C for 30 min or left untreated. Thirteen hours later, a second heat shock of 45°C for 30 min was applied. Cells were allowed to recover after heat shock for 1 h. Relative luciferase activities were calculated as detailed in the legend to Fig. 1. The results are the average of four independent transfections (standard deviations are indicated by *error bars*)

Hageman et al*.* (2007) have shown that the refolding activity of the cytosol, nucleus, ER, and peroxisomes increases in cells that have recovered from a heat shock. Such thermotolerance is due to the increased synthesis of chaperones. To show that the thermotolerance of the refolding in different cellular compartments requires HSF1-regulated gene products, we tested whether dnHSF1 expressing cells can acquire thermostability in the different cellular compartments. As shown in Fig. 3a, in cells that have been pre-heat-shocked, about 45 % of the cytosolic luciferase was refolded within 1 h post-heat shock, while in naïve cells slightly more than 3 % of the pre-heat shock luciferase activity was regained. Expression of dnHSF1 abolished the ability to induce thermotolerance; only 7 % of the cytosolic luciferase was refolded in preconditioned dnHSF1 expressing cells (Fig. 3a). The acquired thermostability of nuclear compartment was less HSF1 dependent as 17 % of the nuclear luciferase was refolded in preconditioned dnHSF1 expressing cells compared with 41 % in normal preconditioned cells (Fig. 3b). Twenty-three percent of the luciferase targeted to the ER was refolded in preconditioned dnHSF1 expressing cells compared with 39 % in normal preconditioned cells (Fig. 3c), showing that the ER is less dependent on HSF1 for gaining thermostability. No chaperones have been detected in peroxisomes, yet luciferase can be refolded in peroxisomes (Hageman et al. 2007; Figs. 1 and 2). Additionally, the refolding activity of the peroxisomes was shown to be increased in cells that have recovered from a heat shock. As shown in Fig. 3d, in cells that have been pre-heat-shocked, about 55 % of the peroxisomal luciferase was refolded within 1 h post-heat shock, while in naïve cells had regained 5 % of the pre-heat shock luciferase activity. Expression of dnHSF1 abolished the ability to induce thermotolerance; only 15 % of the peroxisomal luciferase was refolded in preconditioned dnHSF1 expressing cells (Fig. 3d). These data show that it is the additional synthesis of HSF1-regulated gene products that is responsible for the improved refolding capacity of the peroxisomes in pre-heat shock cells and suggest that refolding of peroxisomal luciferase requires HSF1-regulated chaperones. However, we were not able to rescue the inhibitory effect of dnHSF1 on gaining thermotolerance in peroxisomes by overexpressing chaperones encoded by HSF1 target genes (data not shown).

### HSPB1 promotes peroxisomal refolding

**Fig. 4:**
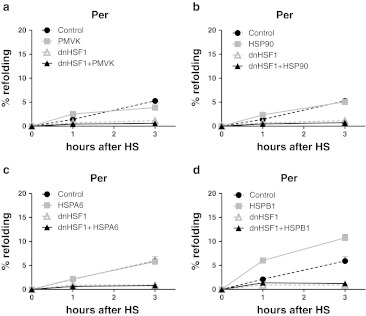
Effect of HSPA6, HSPB1, HSP90AA1, and PMVK on refolding capacities of peroxisomes. Relative luciferase activity of Per-superluc-eGFP in HEK-cDNA5-Per-superluc-eGFP or HEK-dnHSF1-Per-superluc-eGFP cells. Cells were transfected with an expression constructs for PMVK (**a**), HSP90AA1 (**b**), HSPA6 (**c**), or HSPB1 (**d**). The expression levels of the exogenously expressed proteins are shown in Fig. S5. Cells were harvested after heat shock at the times indicated, and the luciferase activity was measured. Relative luciferase activities were calculated as detailed in the legend to Fig. 1. The results are the average of four independent transfections (standard deviations are indicated by *error bars*)

As shown above, cells depend on HSF1-regulated genes for peroxisomal refolding in naïve cells as well as for the ability to develop thermotolerance of peroxisomal refolding. Yet, exogenous expression of the most likely candidates, the HSF1-dependent genes HSPA1A and DNAJB1, did not restore peroxisomal refolding (Fig. 2d). In the refolding assays, cycloheximide is added before the heat shock. The HSF1-regulated function must thus be one that is inhibited by dnHSF1 expression even in the non-stressed state. We have previously shown that the transcript level of only ten genes is significantly (more than two-fold) lower when dnHSF1 is expressed in non-stressed cells (Heldens et al. 2010). Four of these encode (co)chaperone genes, HSP90AA1, HSPA6, DNAJB1, and HSPB1. Of the other six genes (PMVK, KLRG1, CDKL3, KA21, ZNF473, MLH1), only PMVK looks like a possible candidate. The human PMVK sequence contains a putative peroxisomal targeting sequence (Olivier et al. 1999) and was shown to be localized in peroxisomes (Chambliss et al. 1996; Olivier et al. 1999) although others have found PMVK to be mainly cytoplasmic (Hogenboom et al. 2004). A protein–protein interaction study detected multiple interactions of PMVK (Ewing et al. 2007), which might indicate that PMVK could have a second role as a chaperone. We thus tested whether exogenous expression of PMVK, HSP90AA1, HSPA6, or HSPB1 could rescue the decreased refolding capacity of peroxisomes in dnHSF1 expressing cells (the effect of DNAJB1 is already shown above in Fig. 2d). Exogenous expression of none of these four proteins improved peroxisomal refolding in dnHSF1 expressing cells (Fig. 4a, b; note that the extent of peroxisomal refolding shown here is somewhat lower than shown in other figures, presumably due to a slightly harsher heat shock). Intriguingly, in control cells, HSPB1 did improve peroxisomal refolding (Fig. 4d).

**Fig. 5:**
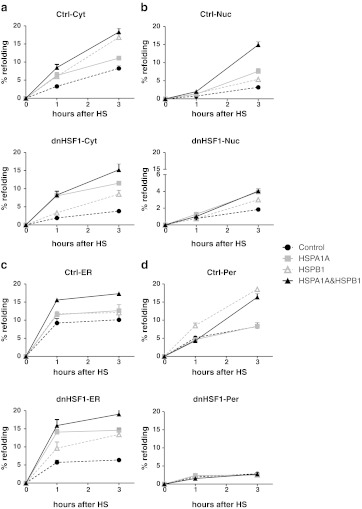
Effect HSPA1A and HSPB1 on refolding capacities of different organelles. Relative luciferase activity of **a** Cyt-superluc-eGFP in HEK-cDNA5 and HEK-dnHSF1 cells; **b** Nuc-superluc-eGFP in HEK-cDNA5 and HEK-dnHSF1 cells; **c** ER-superluc-eGFP in HEK-cDNA5 and HEK-dnHSF1 cells; **d** Per-superluc-eGFP in HEK-cDNA5-Per-superluc-eGFP or HEK-dnHSF1-Per-superluc-eGFP cells. Cells were co-transfected with the luciferase reporter gene and expression constructs for HSPA1A and/or HSPB1 and/or empty vector as indicated. Per-superluc-eGFP cells were transfected only with the expression constructs. Expression levels of exogenously expressed proteins are shown in Fig. S6. Cells were harvested after heat shock at the times indicated, and the luciferase activity was measured. Relative luciferase activities were calculated as detailed in the legend to Fig. 1. The results are the average of four independent transfections (standard deviations are indicated by *error bars*)

HSPB1 cannot refold proteins but can maintain these in a refolding competent state and cooperate with the Hsp70 folding machine, which refolds the HSPB1 substrates. We thus tested whether the lack of an effect of HSPB1on peroxisomal refolding in dnHSF1 expressing cells was due to a lack of HSPA1A. It was not: Exogenous expression of HSPA1A had no effect (Fig. 5). In contrast, in other cellular compartment, an effect of HSPB1 did require HSPA1A. The limiting node in the peroxisomal chaperone network thus differs from that in other compartments.

As exogenous expression of the traditional HSF1 target genes did not restore peroxisomal refolding in dnHSF1 expressing cells, we tested the effect of other chaperone family members as well. Exogenous expression of HSPH1, GRP94, HSPA8, HSP47(SERPINH1), DNAJA1, DNAJB2a, DNAJB2b, DNAJB6, DNAJB8, HSPB5, or HSPB8 had no effect on peroxisomal protein folding (data not shown).

## Discussion

We show here that expression of dnHSF1 not only affects cytoplasmic and nuclear refolding but, unexpectedly, also refolding in the ER and the peroxisomes. The simplest explanation would be that expression of dnHSF1 depletes the cell of cytoplasmic, HSF1-regulated, chaperones. Peroxisomal matrix proteins are imported from the cytoplasm and thought to be folded by the cytoplasmic chaperones. How peroxisomes deal with unfolded proteins is not known. The ER has a retrograde export system for unfolded proteins (ERAD), which are then degraded in the cytoplasm. Peroxisomes are not known to have an export system, but their import system shows a striking similarity to ERAD (Schliebs et al. 2010) with the crucial difference that ERAD exports while the peroxisomal system imports. Perhaps the peroxisomal import system is reversible and can also export when unfolded proteins accumulate in the peroxisomes. These proteins could then either be refolded and reimported or degraded. As we have shown here, peroxisomal refolding is fully dependent upon HSF1 activity and at first glance appears to be equal to, and to be due to, cytoplasmic refolding. However, a closer examination shows important differences. Cytoplasmic refolding in control cells can be augmented by exogenous expression of HSPA1A and is thus limited by the Hsp70 machinery. Refolding of peroxisomal luciferase, in contrast, is limited by co-chaperones. In control cells, it can be increased somewhat by exogenous expression of DNAJB1 which supplies substrate to Hsp70 and by HSPB1, which serves as a holding reservoir for Hsp70 refolding. Hsp70 activity itself is not limiting, as exogenous expression of HSPA1A has no effect on peroxisomal refolding. Peroxisomes thus appear to have Hsp70 activity in excess, while in the cytoplasm, Hsp70 is limiting. A second important difference between cytoplasmic and peroxisomal refolding is that in cells expressing dnHSF1, refolding of peroxisomal luciferase cannot be restored by exogenous expression of any of the (co-)chaperones we tested, while expression of HSPA1A + DNAJB1 fully restored cytoplasmic luciferase refolding. This difference could be due to a spatial restriction of the refolding of peroxisomal proteins. In plants, a membrane-bound DNAJ protein recruits cytoplasmic Hsp70 to the peroxisomal surface (Diefenbach and Kindl 2000), and peroxisomal proteins could thus be refolded locally on the cytosolic side of the peroxisomal membrane rather than dispersed in the cytoplasm. An alternative possibility to cytoplasmic refolding of peroxisomal proteins is that peroxisomes do import chaperones under conditions of proteotoxic stress. It has been shown that peroxisomes import unfolded BSA along with HSPA8 (Hsc70) (Brocard et al. 2003). A transient accumulation of chaperones in peroxisomes during proteotoxic stress could then ensure refolding. Whatever the mechanism of peroxisomal refolding is, it requires a crucial component of which the function is somehow dependent upon HSF1-regulated gene expression. What that component is, is unknown; none of the chaperones tested in our experiments restored peroxisomal refolding in dnHSF1 expressing cells. Expression of dnHSF1 did not impair import of peroxisomal-targeted eGFP–luciferase: In cells cultured for 12 days with continuous expression of dnHSF1, the localization of peroxisomal eGFP–luciferase was not affected (Fig. S7).

The ER has its own set of chaperones. Except for SERPINH1 (Hsp47), the synthesis and level of the ER chaperones is not regulated by HSF1. Yet, we do find that expression of dnHSF1 also results in a 50 % inhibition of refolding of ER-targeted luciferase. We cannot rigorously exclude that some of the ER-targeted luciferase is mislocalized in the cytoplasm. However, if that was the case, we would expect that exogenous HSPA1A would improve ER luciferase refolding in control cells just as it improves cytoplasmic luciferase refolding. It did not. The most likely explanation is that somehow lack of cytoplasmic chaperones clogs up the ER refolding machinery. Cytoplasmic domains of integral membrane proteins are folded by cytoplasmic chaperones. In addition, cytoplasmic Hsp70s are implicated in ERAD (Nakatsukasa et al. 2008; Oyadomari et al. 2006). In our hands, a lack of cytoplasmic chaperones due to dnHSF1 expression could not be relieved by exogenous expression of HSPA5, the ER resident Hsp70. If anything, exogenous expression of HSPA5 inhibited post-heat shock refolding. Curiously, HSPA5 was effective in preventing denaturation during heat shock, and HSPA5 was the only exogenously expressed chaperone that did not show a direct correlation between protection during heat shock and of post-heat shock refolding, suggesting that in this case these are distinct processes.

Previously we showed that the activity of the glucocorticoid receptor, a client of both the Hsp70 and the Hsp90 machines, could be restored in dnHSF1 expressing cells by exogenous expression of DNAJB1 but not HSPA1A (Heldens et al. 2010). In contrast, exogenous expression of DNAJB1 did not restore refolding of luciferase; HSPA1A is required as well. Which node of the chaperoning network is critical thus depends on the substrate tested and the compartment in which the substrate is located. Increasing expression of (co)-chaperones to compensate for the loss of HSF1-regulated chaperones in aging cells thus needs to be tailored for specific substrates.

## Electronic supplementary material

Below is the link to the electronic supplementary material.

ESM 1 (DOC 30 kb)

ESM 2 (EPS 3547 kb)

ESM 3 (EPS 9717 kb)

ESM 4 (EPS 4204 kb)

ESM 5 (TIFF 1838 kb)
