# Supplementary material for: Protein refolding in peroxisomes is dependent upon an HSF1-regulated function
Source: Cell Stress Chaperones. 2012 Apr 5;17(5):603–13. doi: 10.1007/s12192-012-0335-5 (PMC3535170; doi:10.1007/s12192-012-0335-5)
Supplement: Supplementary file 1 — (DOC 30 kb) [file 12192_2012_335_MOESM1_ESM.doc]

**Fig. S1: Effect dnHSF1 and HSPA1A or DNAJB1 expression on luciferase denaturation in different organelles**

Luciferase activity directly after HS of A) Cyt-superluc-eGFP in HEK-cDNA5 and HEK-dnHSF1 cells; B) Nuc-superluc-eGFP in HEK-cDNA5 and HEK-dnHSF1 cells; C) ER-superluc-eGFP in HEK-cDNA5 and HEK-dnHSF1 cells; D) Per-superluc-eGFP in HEK-cDNA5-Per-superluc-eGFP or HEK-dnHSF1-Per-superluc-eGFP cells. Cells were co-transfected with the luciferase reporter gene and expression constructs for HSPA1A and/or DNAJB1 and/or empty vector as indicated. Per-superluc-eGFP cells were transfected only with the expression constructs. Cells were harvested directly after heat shock and the luciferase activity was measured. Luciferase activities were calculated relative to those in non heat shocked cells transfected and cultured in parallel. The results are the average of four independent transfections (standard deviations are indicated by error bars).

**Fig. S2: Effect of exogenous expression of PMVK, HSP90AA1, HSPA6 or HSPB1 on denaturation of peroxisomal luciferase**

Luciferase activity directly after HS of Per-superluc-eGFP in HEK-cDNA5-Per-superluc-eGFP or HEK-dnHSF1-Per-superluc-eGFP cells. Cells were transfected with an expression constructs for HSPA6, HSPB1, HSP90AA1 or PMVK. Cells were harvested directly after heat shock and the luciferase activity was measured. Luciferase activities were calculated relative to those in non heat shocked cells transfected and cultured in parallel. The results are the average of four independent transfections (standard deviations are indicated by error bars).

**Fig. S3: Effect of exogenous expression of HSPA1A and HSPB1 on luciferase denaturation in different organelles**

Luciferase activity directly after HS of A) Cyt-superluc-eGFP in HEK-cDNA5 and HEK-dnHSF1 cells; B) Nuc-superluc-eGFP in HEK-cDNA5 and HEK-dnHSF1 cells; C) ER-superluc-eGFP in HEK-cDNA5 and HEK-dnHSF1 cells; D) Per-superluc-eGFP in HEK-cDNA5-Per-superluc-eGFP or HEK-dnHSF1-Per-superluc-eGFP cells. Cells were co-transfected with the luciferase reporter gene and expression constructs for HSPA1A and/or HSPB1 and/or empty vector as indicated. Per-superluc-eGFP cells were transfected only with the expression constructs. Cells were harvested directly after heat shock and the luciferase activity was measured. Luciferase activities were calculated relative to those in non heat shocked cells transfected and cultured in parallel. The results are the average of four independent transfections (standard deviations are indicated by error bars).

**Fig. S4: Exogenous expression of HSPA1A and DNAJB1**

Levels of exogenous expression of HSPA1A and DNAJB1. Expression plasmids for the (co-)chaperones indicated were transfected into either A) HEK-cDNA cells, HEK-HSF379, B) HEK-cDNA5-Per-superluc-eGFP or HEK-dnHSF1-Per-superluc-eGFP cells and expression of dnHSF1 was induced by adding doxycyclin. Protein levels were determined by western blotting and staining with the corresponding antibody (see ‘‘Materials and methods’’). γ-tubulin was used as a loading control.

**Fig. S5: Exogenous expression of HSP90AA1, HSPA6, HSPB1 and PMVK**

Levels of exogenous expression of different (co-)chaperones. Expression plasmids for HSP90AA1, HSPA6, HSPB1 and PMVK were transfected into either HEK-cDNA5-Per-superluc-eGFP or HEK-dnHSF1-Per-superluc-eGFP cells and expression was induced by adding doxycyclin. Except for HSP90AA1, of which expression is constitutive. Protein levels were determined by western blotting and staining with the corresponding antibody (see ‘‘Materials and methods’’). Note that in the case of HSPA6 antibody to the V5-tag carried by the exogenous proteins was used; the endogenous protein is thus not detected. β-actin was used as a loading control.

**Fig. S6: Exogenous expression of HSPA1A and HSPB1**

Levels of exogenous expression of HSPA1A and HSPB1. Expression plasmids for the (co-)chaperones indicated were transfected into either A) HEK-cDNA cells, HEK-HSF379, B) HEK-cDNA5-Per-superluc-eGFP or HEK-dnHSF1-Per-superluc-eGFP cells and expression was induced by adding doxycyclin. Protein levels were determined by western blotting and staining with the corresponding antibody (see ‘‘Materials and methods’’). β-actin was used as a loading control.

**Fig. S7: Effect of dnHSF1 expression on the localization of peroxisomal targeted luciferase.**

## HEK-dnHSF1-Per-superluc-eGFP cells (cultured without doxycycline or cultured in the presence of doxycycline for 12 days) were plated on **poly*-*L*-*lysine (0.001%, Sigma-Aldrich) coated coverslips**. Cells were fixed in 4% paraformaldehyde. All images were obtained by fluorescence microscopy using the DMRA fluorescence microscope with a COHU CCD camera and QFluoro 1.2 software.
