# Supplementary figures and images for: Protein refolding in peroxisomes is dependent upon an HSF1-regulated function
Source: Cell Stress Chaperones. 2012 Apr 5;17(5):603–13. doi: 10.1007/s12192-012-0335-5 (PMC3535170; doi:10.1007/s12192-012-0335-5)

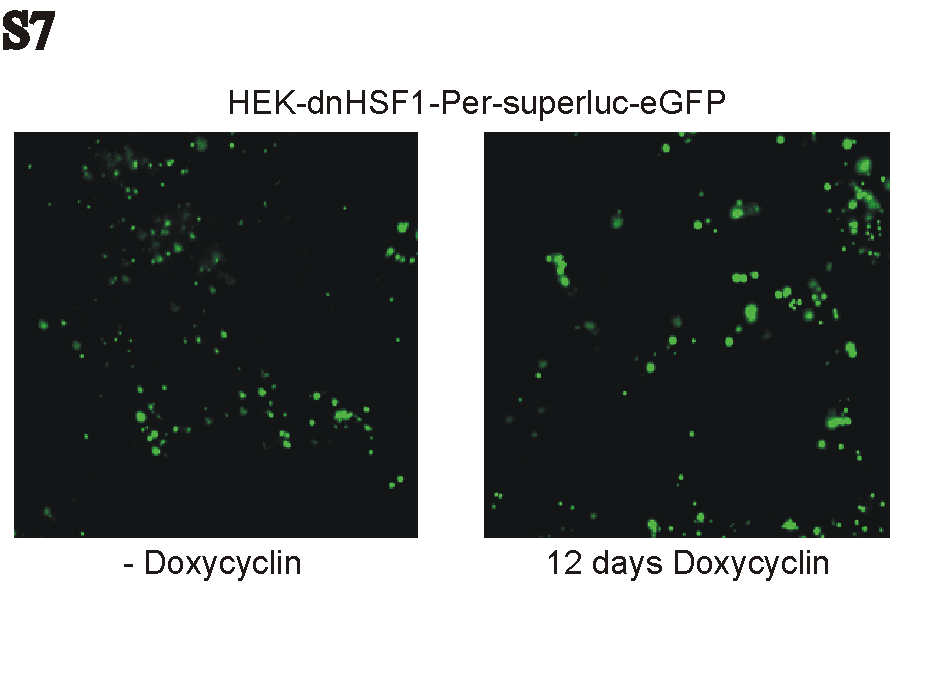

Supplement: Supplementary file 5 — (TIFF 1838 kb) [file 12192_2012_335_MOESM5_ESM.tif]
